# Development of a behaviour change intervention to promote sanitation and latrine use in rural India

**DOI:** 10.1186/s12889-023-17061-0

**Published:** 2023-11-06

**Authors:** Usman Talat, Luke Ravenscroft, Ivo Vlaev

**Affiliations:** 1 Alliance Manchester Business School, University of Manchester, Manchester, M13 9PL UK; 2 Behavioural Insights Team, 4 Matthew Parker Street, London, SW1H 9NP UK; 3 Warwick Business School, University of Warwick, Scarman Rd, Coventry, CV4 7A UK

**Keywords:** Latrine use, Open defecation, Rural perceptions, Behaviour change intervention

## Abstract

**Background:**

Across developing countries poor sanitation is associated with disease often found widespread in rural populations.

**Objectives:**

This objective of this study was to conduct a formative research and feasibility evaluation of the behavioural intervention designed to improve latrine use in rural India.

**Methods:**

Study conducted in four villages of Rajasthan, where latrine use is low and open defecation may spread disease. To identify the intervention a literature review was conducted, a survey of 497 households, and focus groups in village households (8–10 women and children). Seven focus groups with 63 women were conducted. Based on the survey results, the behaviour change intervention is developed utilising the Capability-Opportunity-Motivation-behaviour model and MINDSPACE framework. One intervention component involves psychological aspects that engage villagers through a pledge; the other component is provision of small incentives to facilitate latrine use. Feasibility and acceptability of the intervention was examined in the study population. The 30-day intervention was delivered to women in 38 randomly selected households who despite having a functional latrine did not use it. Thematic analysis, binary logistic regression analysis and feasibility evaluation of the intervention conducted. Post-intervention feedback from 22 participating households was obtained.

**Results:**

The piloted intervention was feasible and so a revised design is offered. Results driving this evaluation include barriers identified, and used to improved intervention design in the current study. Village authority figures influenced behaviours across the villages and so did factors of convenience (β = 5.28, *p* < 0.01), relief (β = 5.49, *p* < 0.01), comfort (β = 2.36, *p* < 0.01), Construction cost (β=-1.98, *p* < 0.01) and safety (β = 2.93, *p* < 0.01) were significant concerns associated with latrine use in the context of prevalent OD in the region. The logistic regression baseline model for the dependant variables indicated a significant increase in latrine use. Based on the feasibility study, the intervention is refined in several ways.

**Conclusions:**

Our theory-driven approach improves latrine use in Rajasthan and offers a useful tool to facilitate hygiene behaviour.

**Supplementary Information:**

The online version contains supplementary material available at 10.1186/s12889-023-17061-0.

## Introduction

Poor sanitation is associated with the spread of serious disease in society, found to be pervasive throughout developing countries. Amongst countries suffering from this problem, India has to bear the brunt of the impact on its rural population with associated levels of high infant mortality and contagion [1, 2]. Villagers experience psychosocial stress due to the risk of physical attacks alternative sanitation practices including open defecating carry. These include serious attacks including rape and molestation, as well as hazards such as snake and mosquito bites, and foot injury incurred due to lack of adequate footwear [3–5]. Latrine use offers a safer alternative that improves sanitary conditions and curbs the spread of disease in populations across villages [6]. Diseases cause an estimated 22% of non-neonatal child deaths associated with Open Defecation (OD) practices [7].

The Swachh Bharat Abhiyan in 2014 and Nirmal Bharat Abhiyan in 2012, relaunched India’s Total Sanitation campaign 2000 with the goal of improving coverage by incentivising the population of the country and investing in building infrastructure including latrines and improved access to facilities. Coverage includes household access to latrines and sewerage [8] but is not necessarily indicative of increased latrine use by a population. Furthermore, reporting latrine installation numbers notwithstanding quality, remains simpler than recording behaviour at population level that exhibits variation across India [6, 9, 10]. One example is the situation where the Bihar state of India reported 48% coverage, by contrast in rural Kerala 100% was reported, and in rural Odisha fewer than 50% report latrine use only [1, 11].

Dysfunctional infrastructure can form barriers for those who may otherwise take up latrine use [3, 6, 8, 12] on this front working latrines that are well maintained can encourage better sanitation practices [1, 13]. Poorly constructed latrines tend to be lower-quality and less appealing to users. Studies also highlight poor water quality and availability as a barrier to use. Latrines lacking water supply and septic tank structures induce avoidance behaviours [14]. Other structural features include the presence of complete structures (a roof and side walls) to improve privacy, a drainage system and ease of access [15]. A major factor in latrine uptake is culture [8]. Specifically, choosing the alternative of OD over latrine use is a preference influenced by longstanding habits, rituals, caste, lifestyle routines, gender, age and marital status [3] with mortality at over 10% of India’s population. In rural areas of India, upper caste individuals usually have better access to latrine facilities and water, by contrast individuals from lower classes face barriers. The norm is that individuals and families of lower caste prefer defecating outdoors because it can be more practical and convenient [3, 16].

The implementation of the Total Sanitation Campaign in India also highlighted the importance of caste. An example is the building of school latrines in villages where lower caste students were forced to clean the toilets [8, 17, 18]. Such acts discourage latrine use because of consequent negative perceptions. It has been noted that if women had decision-making power across households, more latrines would likely be used [19]. Some women eat less so they do not have to go out at night to defecate [20], which has dire effects on their health. Studies also found that men are often not responsive to the challenges women face in practicing OD [21]. In this regard, marital status is another factor in latrine use and related sanitary practices. A study reported that in parts of rural India, before agreeing to marry a husband, women showed a strong preference for the ownership of latrines [22].

Additionally, significant behavioural factors associated with latrine use include education and awareness. A study in rural Timor found that educated individuals were most likely to use latrines [23] because they were aware of the benefits of latrine use and harms of OD practices. A study of rural coastal Odisha in India reported that during their formal education, hostel students exposed to latrines would likely adopt the habit. They were also more likely to be high caste individuals with the financial means to obtain formal education [3]. Despite pedagogical factors, OD may persist because in some cases, it may be deeply embedded as a social practice in rural communities.

In north India’s rural areas despite access to latrines, some villagers preferred defecating openly because they saw it as more comfortable and convenient [24]. Another reason to explain preferences and sanitary choices was found by a study at a village in Nepal where the practice enabled socialization. Friends had used open fields growing up. It was a time in the morning to share stories, neighbourhood gossip and to plan the day [25]. Literature shows that some interventions to improve adherence target infrastructure, some target socio-cultural behaviour and some interventions aim to target a combination of these two factors [3, 8, 10, 25]. A study commissioned by the world health organization advocates the need for interventions [8].

The current study developed and conducted a formative evaluation of the intervention to improve latrine use in Udaipur Rajasthan, India. A focus was established on understanding barriers to latrine use, behavioural theory informed intervention feasibility and prospective solutions to increase latrine use.

1. What are the barriers to latrine use in this part of India?
2. Can a theory-driven behavioural intervention be co-designed and does a preliminary examination show that the intervention is feasible and acceptable?
3. What solutions could help increase latrine use?

## Method

### Study design

The study design includes stages with surveys and focus groups to elicit perceptions concerning barriers to latrine use. Informed by this understanding the development of theory-informed intervention is presented, further revised through evidence and expert consultation and feedback procedures outlined in this section. Table 1 depicts this as steps.

**Table 1:** Steps for the development of the behaviour change intervention

	Method	Use	Aim
**Step One**	Surveys and focus groups	To understand barriers to Latrine use	Inform intervention design choices
**Step Two**	Theoretical development	Theory guided practice	Design grounded in theory
**Step Three**	Evidence via expert consultation/feedback.	Feedback from stakeholders in actual setting	Revision of intervention design

Qualitative and quantitative data was collected to determine reasons for poor latrine use, and to inform design choices to make the intervention more feasible. A multi-village survey was conducted using tablets, on which surveyors were trained. Quantitative data collected through software Kobocollect installed in a mobile phone (see Additional file 1: Appendix 6). The purpose of the project was explained to villagers under guidance and cues from experts including researchers, Seva Mandir employees, and the village head known as *the Sarpanch*.

The four villages - and their adjoining hamlets are located in Udaipur district. It was found that houses were semi-pukka or pukka^1^ in the main village. However, most houses in the hamlets were kutcha (composed of materials including structures made of clay, brick, cement, and organic mixtures of stone). 228 out of 497 sample households were below the poverty line and 20% of the total sample covered did not have latrines. The quantitative survey covered all individuals across 497 households in total, with 404 households from tribal communities. The qualitative survey included observations, focussed group discussions (FGDs) with women, men, their children (under 16) and youth of the villages, including a mix of boys and girls between the ages of 16–19 years. Discussions were held in groups of 8 to10, and in-depth interviews with women.

The barriers identified through the qualitative survey were discussed to corroborate findings and new barriers if any, were noted. The FGD elicited responses concerning the notion of small improvements such as buckets, soap and cleaning equipment. The assertion is that adding the provision of items to the intervention would encourage use of toilets in so far as this removes barriers to use. Village leaders including the Sarpanch (i.e. village head) and influencers such as ASHA (accredited social health activist) workers and Anganwadi workers were also consulted to understand their efforts towards ending OD practices. A meeting was held with the Sarpanch where the fact the district collector had taken a lot of interest in the issue of defecation had been raised by a ward member. To further facilitate information gathering, data was also collected on landholding patterns, availability of water sources and cost-sharing for latrine construction across districts. This furnished structural context for our interpretations. Questions were also posed around perceptions of latrine use, reasons for OD preference, perception regarding the extent of latrine use by others in the community (See Additional file 1: Appendix 2 and 4, pilot plan and intervention script).

*Theory* – This stage identified design and implementation of the pilot intervention in villages located in the Udaipur district of India (villages see Table 3). A behavioural intervention design based on systematic behaviour change theory was selected. This is the COM-B framework that posits human behaviour as an interacting system, where capability (physical and psychological), opportunity (social and physical) and motivations (automatic and reflective) interact to generate behaviour [26]. The adjusted model provides a frame for our research.

In our adapted model, the behaviour of concern is adherence to latrine use, whilst seeing open defecation as detrimental in implicating poor sanitation and hygiene behaviour that spreads disease. Our intervention builds capability of villagers (physical ability through provision of items, see Additional file 1: Appendix 1 items) and psychological capacity though a pledge of commitment.

**Fig. 1:**
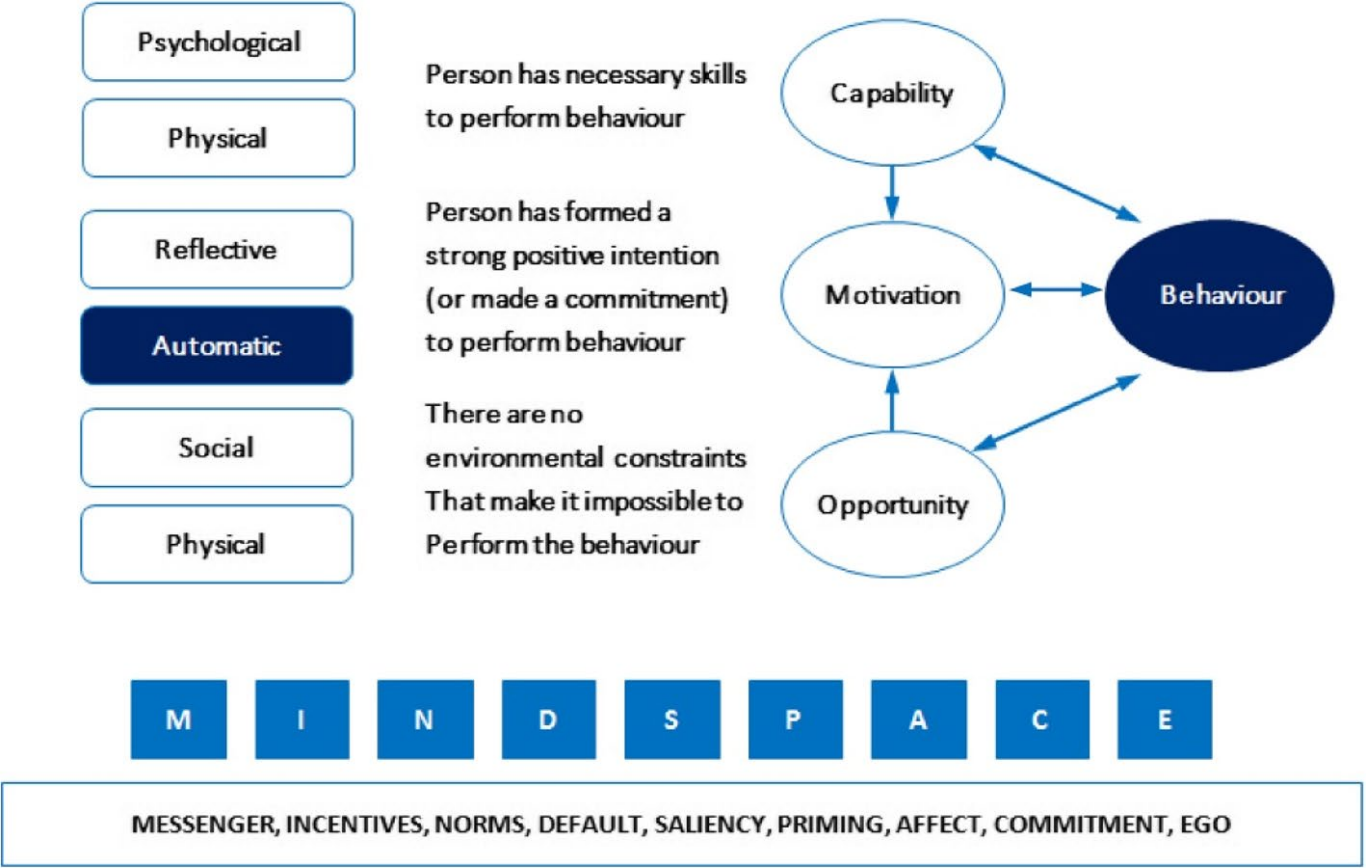
Extended COM-B theory including the MINDSPACE framework of automatic motivations

Drawing on the MINDSPACE framework that concerns social dynamics, human psychology and behaviour, our underlying assertion relies on automatic behaviour tendencies. The framework (Fig. 1) consists of mechanisms of behaviour change denoted under the MINDSPACE acronym – these are messenger, incentives, norms, saliency, priming, affect, commitment, Ego. These robust behaviour change mechanisms predominantly operate through automatic motivational processes. This framing further sharpened our focus [27, 28]. The framework provides guidance on tools to change behaviour within the broader parameters of the COM-B model (refer to Additional file 1: Appendix 1 commitment explanation). MINDSPACE underlines the COM-B structure, and explains automatic behaviours amenable to intervention conditions, with the aim of changing latrine use behaviour significantly in rural India.

In this respect, the use of theory was an advantage in conceptualising our approach and integrating understanding of hygiene behaviour and habit formation. It was found that the key barriers to treatment adherence were the absence of adequate positive feedback, especially in the initial stages, lack of tangible benefits / rewards for adherence, long term impact of non-adherence and lack of routine [26, 27]. Our solution is a commitment tool, which aimed to facilitate the creation of a new habit.

### Inclusion criteria

▪ The sample selected was representative of demographic indicators such as caste, economic status (whether above poverty line (APL) or below poverty line (BPL)) and religious demographics in Udaipur.
▪ Villages where defecation in the open is high and latrine use is low, including villages declared OD-free by the government.
▪ All willing members of the HH (household) will be included.
▪ Kucha and pukka HH.
▪ A working toilet located in the HH.

### The socioeconomic characteristics

The research area is located in Udaipur District of Rajasthan and is largely tribal. The main forms of livelihood in the area include agriculture, farm labour, livestock rearing, dairy, skilled work and private businesses. Levels of access to water vary and while some households have their own water sources through tube-wells or wells, other villagers depend on public sources. Hand pumps are available though they tend to run dry during peak summers. Eco-san, septic tank and twin pit latrines are prevalent in the area. Most of the latrines have been constructed in the last two years (see Additional file 1: Appendix 5). The SBM was anchored by the Department of Rural Development and Panchayati Raj in the Rajasthan area.

*Developing the Variables* – Independent variables were developed through a series of steps. This involved reviewing the literature, discussions with Seva Mandir representatives trained to collect data, comprehensive observation of on-site infrastructure related to access and ease of use, the use of templates for standardisation of variables and construction of items that collectively represent various aspects of the independent variables that were discussed and finalised. The dependent variable is latrine use in this model. The variables in the study represent barriers targeted using the provision of incentives in the intervention design.

During the preparatory phase of the project, members of village panchayat (council) also participated in most discussions. Thus, communities were directly involved in developing the content (questionnaires, measurement tools) of the proposed research.

### Data analysis

Qualitative data analysis involved critical discussions across focus groups, conducted with the various village heads known as “the Sarpanch”. Participants include community workers who brought a grounded perspective during discussions. The data from the focus groups was analysed using thematic analysis. Groups consisted of expert academics, who in sessions with practitioners (social workers with NGOs) and benefitting from the Sarpanch’s guidance; identified barriers that seem meaningful and important in the decision to use a latrine.

To quantitatively evaluate barriers to adherence post intervention implementation, the survey data was cleaned, reviewed and binary logistic regression was performed using statistical software [SPSS 25 v.25]. Measures encoded “other than only latrine use” into 0 and “only latrine” into 1 as nominal categories. A range of independent variables were selected, these were about household and village infrastructure, financial constraints and social status, logistics and access, including convenience and behavioural responses. Including a range of emotions and perceptions of latrine use (see Additional file 1: Appendix 3 - list ordinal and nominal variables). Statistical procedure includes the omnibus test.

## Results

### Formative evaluation of intervention

#### Barriers to latrine use

Results of implementing our intervention through a revised binary logistic regression are presented. These results show whether the intervention worked in identifying barriers to latrine use. The “ Discussion” section follows with regard to design and theory guided intervention design, application and subsequent evaluation for feasibility.

The omnibus test indicates an improved and significant fit of the revised model to data (*p* < 0.01). In the category only latrine use correctly classified (*n* = 204) and misclassified (*n* = 12). In the category other than only latrine use correctly classifies (*n* = 174) and misclassifies (*n* = 8). Overall, an accuracy of 95% reported. The logistic regression baseline model for the dependant variables indicated “Only latrine” use likely to be correct 53.3% of the time. Hygiene behaviour that is convenient and provides desired relief is a significant factor associated with the likelihood of latrine use. If latrines are not complete in construction due to costs, then convenience of OD appears attractive to villagers. Survey results show that BPL families are more likely to use a latrine compared with APL families, indicating social status as a significant influence in sanitary behaviour. In terms of infrastructure barriers, the presence of latrine inside the house (latrine location) increased the likelihood of use. Increase in the cost of constructing the latrine and monthly expense per household were factors likely to increase use of a latrine. Comfortable latrines increased the likelihood of their use, and villagers who saw latrines as less convenient were less likely to use them. Despite the fact that nearly 80% households had latrines, the observed latrine use was only 37%^2^.

 Table 2 summarizes the logistic regression analysis results, these include details for significant independent variables measured in associated with likelihood of latrine use.

**Table 2:** Logistic Regression Analysis Result (significant variables) (sample *n *= 398)

Variable	β	Exp (β)	*P*
APL/BPL	− 0.1.58	0.2	0.02
HHE	1.71	5.56	0.05
Latrine Location	1.69	5.46	0.33
Construction Cost	-1.98	0.13	0.00
Comfort	2.36	10.62	0.00
Convenience	5.28	197.31	0.00
Social Pressure	1.67	0.18	0.17
Perception	-0.851	0.42	0.45
Happy	1.5	4.5	0.94
Safety	2.93	18.78	0.01
Relief	5.49	242.8	0.00

Cut off for Significance *p* < 0.10 - The Omnibus test looking for effects of our independent variables is highly significant at (*p* < 0.01). Using the Nagelkerke’s R suggests that the model fits 89.7% to actual results. This explains a high percentage of variation in results. The Hosmer and Lemeshow Test gives a good fit to the data (*p* = 0.89). Correctly classified outcomes of 95% of the cases.

*ABL/BPL* Identifies economic status, caste and religious demographic, *HHE* Monthly Household expense on latrine, *Latrine Location* Position of latrine in Household, *Construction Cost* Construction cost of latrine, *Comfort* Place of defecation family members found most comfortable, *Convenient* Find latrine use convenient, *Social Pressure* Social pressure to use latrine, *Perception* Perception of villagers use of latrines, *Happy* Happiness in thinking about latrine, *Safety* Feeling safe thinking about latrine, *Relief* Feeling relief thinking about latrine

In terms of social influences, HH without latrines were facing pressure by others in the village to use the new techniques. Villagers who perceived that a high proportion of households were using latrines across the villages, were themselves less likely to use latrines. It was found that villagers who felt happiness, relief and safety when thinking about latrines were more likely to use them as well.

Despite toilets present in HH, women in the villages found it difficult to haul enough water, a task men will not do. For example, in village C, one woman said that while toilets are convenient and safer, lack of water availability was a key barrier to her latrine use. She said that she practiced OD due to this water shortage.

Through fieldwork in the villages, focussed group discussions and commitment engendering visits to HH for interviews, a range of barriers and behaviours were identified. These impeded the use of toilets and provided reasons for the continuing practice of OD. Scarcity of water was a barrier in all four villages. Table 3 summarizes the water-related barriers encountered during the research fieldwork.

**Table 3:** Water-related barriers encountered during the research fieldwork

Setting	Barriers (water related)
Village – A	▪ Water scarcity, only women bring water, so less available. ▪ Water supply was an issue, only improved in the past year. ▪ Water storage was identified as an issue. Insufficient storage.
Village – B	▪ Scarcity of water discouraged latrine use, and led to OD. ▪ Only women brought the water and managed latrines. They often could not manage.
Village - C and Hamlet C	▪ The toilet structure impractical for storing water and hard to use. ▪ Village well and other water sources dried up due to heat. ▪ For some households a toilet in the household was a sign of upward mobility, but this was curtailed by scarce water availability
Village - D and Hamlet D	▪ Sanitation duties including hauling water was too to manage. ▪ Hand pumps near households encouraged latrine use of residents.

In relation to the infrastructure issues presented in Table 2, some Eco San toilets were found in the two villages. Design flaws and structural deficiencies were also present. In the villages, the quality of latrines was especially poor where private contractors had constructed the toilets (Additional file 1: Appendix 5).

In order to construct toilets, villagers were told to use their own funds, reimbursable at a later date by the government. But many villagers ran out of funds and were consequently left with incomplete latrines. This left them without government reimbursement, obtainable only by completing the construction. Thereby, villagers were placed in a hopeless situation. Also, age was found to be a factor in uptake. Specifically, older villagers found latrine use difficult. Likewise, those with illnesses also struggled.

Discussions revealed that awareness of the benefits of latrine use and the health and hygiene hazards associated with OD was almost non-existent in the villages. Absence of this awareness meant it was easier to prioritise other water-related activities including cooking, drinking and cleaning. There was also lack of awareness about sanitation procedures to do with regular pit emptying. Most villagers had not been educated about hygiene procedures, which may likely become an issue going forward. Literacy across the villages for Rajasthan are reported at 79% male, 52% female and 66% overall, among the lowest in India [29–31]. Other studies reported literacy at 66.1% [32], 62.71% [33] and 67.10%-below the national average of 74.04% [34].

In the villages, there were unused government constructed toilets, which further reflected our concern for low latrine use and the preference for OD by villagers. Informal attempts to raise awareness were found on wall writings across villages, which talked of the need to use latrines (Additional file 1: Appendix 5). This highlighted that emotions like shame were present regarding poor latrine use and participation in OD. And fear of the Sarpanch (head of the village) may also be associated with higher latrine use and less defecation in the open. Whether the village Sarpanch can influence villagers and what qualities as influence, presents area for further inquiry and development research, with potential to inform policy level actions.

In brief, our findings were:

(i) Capabilities and Opportunities: 20% of households were still without a latrine. Observed usage was 37% and varied across the four villages (survey answers were matched with observations of whether the latrines looked as if they had been used). Only 53% reported that all members of the household used the latrine (35% continued to defecate in the open, and 12% did both). Usage was generally higher amongst women for reasons including convenience and safety, and amongst wealthier households. Older people who used latrines often did so due to restricted mobility as well as age and health conditions. Another barrier related to capability was cleaning: respondents had limited awareness of how to clean their latrines daily, and had unrealistic ideas concerning the costs involved. Opportunity (i.e., infrastructure) was a key barrier to latrine usage; water (75%), maintenance cost (45%) and pit size (36%) were the most commonly cited concerns.
(ii) Reflective Motivations: A prominent issue was latrine ‘experience: 31% believed that the latrine was dirty and unsuitable to be placed within or even near their home; smell was a concern for 29%; and comfort for 15%. Householders also felt that the latrines were a long way from being ‘their’ toilets, this indicated a sense of lack in ‘ownership’.
(iii) Automatic Motivations: People were ‘habituated’ to defecating in the open, and considered the idea of using a latrine alien, with some individuals reporting feelings of claustrophobia and disgust.

### Feasibility and acceptability of the intervention

Post formative research, the question was asked “Can a theory-driven behavioural intervention be co-designed and does a preliminary examination show that the intervention is feasible and acceptable?” The intervention was developed by reviewing the literature and applying the COM-B model to address the psychological and practical barriers of latrine use, found in the formative research. The intervention comprises three elements (see the Additional file 1: Appendix).

(i) Capabilities and Opportunities: In discussion with household members barriers related to psychological and physical capabilities were explained and social opportunities were exemplified with for instance demonstration of physical benefits. Educating the villagers (e.g., explaining how to use and clean the latrine) addressed identified barriers and provided the chance to correct false beliefs (e.g., about water supply and costs involved). Overall, pedagogy improves capabilities and enables people in carrying out sanitary behaviours [35].
(ii) Reflective Motivations: In reflecting on changing sanitary behaviours, negative experience was a crucial self-reported factor by villagers. Through the intervention households were able to choose improvements to this experience. Each household choose two bespoke ‘small improvements’ from a pre-selected list (e.g., air freshener, light, water tool/bucket) to make and install inside their latrine; thereby improving the ‘experience’ of using the latrine and also increasing the sense of ‘ownership’. The second component involves provision of small incentives in terms of tangible materials to promote latrine use, these were selected based on barriers identified. Tangibles include items such as buckets with mugs, soaps, brushes, toilet fresheners, solar lights and windows / ventilation for toilet. The team *measured latrine use* through a combination of the following methods:
  1. Paint was used around the latrine pit where people put their feet. Follow up visits at regular intervals of 3 months involved evaluating how worn out they appeared.
  2. Dipsticks that fit around toilet pipes which would be used as a measurement tool at the start and end of a definite period (roughly 6 months).
  3. Toilet Soaps / Scrubbing Bubbles were inspected to assess wear away, as people use the toilet. This reflects extent of use. A limitation is that soap might be used for general hygiene. Here the commitment and pledge induced volition and will to participate is a biasing factor (see Additional file 1: Appendix 1).
(iii) Automatic Motivations: The small improvements made should also tackle feelings of claustrophobia (e.g., light, window) and disgust (e.g., air freshener, water tool). Specifically, the provision of soap and other hygiene items for instance fundamentally facilitates change in perceptions about the difficulty of using latrines. This is encapsulated in variables that are itemised, including convenience, cost, comfort or emotional responses - see Table 2. When combined with a pledge based on MINDSPACE principles and treatment adherence tendencies, the intervention furnishes double-sided impact on perceptions of latrine use and its overall attraction in terms of family level commitment.

The most important reported automatic motivational barrier was habitual open defecation. Commitments are known to break old habits and initiate new habits [36]. Psychological commitment occurs because individuals (automatically) seek to be consistent with their public promises to stick to specific goals or plans. Householders were asked to commit by signing a behavioural contract that contains the statement *“This household is committed to using our toilet for next 28 days because we care about the health of the children of our village”.* The contract was printed on a poster, which will be displayed somewhere prominent in their home to increase its ‘salience’ *(we attend to what is novel and seems relevant to us)*. Expected duration deemed a sufficient period to develop a new everyday habit is four weeks [36].

The intervention utilizes several commitment techniques that tap into relevant psychological processes by utilizing Messenger, Incentives, and Ego mechanisms of change from the MINDSPACE framework [26]. The poster also contained a photo of the family in order to increase the salience of the long-term goal, which in this case was the health of the family. The poster also showed a calendar and provided ‘incentives’ to reinforce the new habit – these were ‘smiling face’ stickers [37]. Women were instructed to put a sticker on the calendar only after observing everybody in the family using the latrine on each day, a sense of responsibility in keeping count. In order to enhance the commitment, the poster showed a picture of human eyes which motivates compliance by subconsciously ‘priming’ (activating) ideas about ‘being watched’ [38] (Female eyes were posted because the women took on the stewardship of our intervention).

### Feasibility study

As part of the funded formative research, the intervention was examined concerning whether it is feasible and acceptable in our study population. Seven focus groups were conducted with 63 women and the intervention was delivered to women in 38 randomly selected households, who despite having a functional latrine, did not use it. The intervention was delivered in Hindi, according to a standardised protocol (see English version in the Additional file 1: Appendix). Following the intervention, feedback was obtained from 22 participating households. It was found that 79% of households chose to make the 30-day commitment to use their latrines. The most preferred component of the intervention was reported as the discussion and selection parts, where small improvements items were identified.

The sample families were selected through random sampling, where the criteria of selection were non-usage of toilet with a functional toilet available in households. There was great pressure at the time from the government at local levels. Policy focus was on pressing improvements in latrine use around rural parts of India.

In such policy context, the type of intervention chosen was based on some input that was interpreted in discussions held with families in households, building from the ground up. Each household selected has a fully constructed toilet, but were not using it. A discussion was held about the intervention elements, their purpose and the rationale of use. Discussions described details of the process and explained the acceptability and feasibility of the pledge and the poster. With clarity and approval from families, the intervention was undertaken with informed consent, thereby eliciting full cooperation and willing participation. Once the family was fully informed about the nature of the intervention and consent was obtained, the pilot was implemented.

### The APEASE framework

Intervention refinement involved brainstorming, where the (APEASE) Affordability, Practicality, Effectiveness/cost-effectiveness, Acceptability, Side-effects/safety and Equity) criteria was adopted for guidance. The intervention design was iteratively reviewed by respondents from the community, and based on their reactions the final components of interventions evolved into a dual-component tool. The intervention tool was finalised under discussion amongst the Seva Mandir and academic experts in the field.

In addition, Seva Mandir builds and nurtures grassroots level institutions called Gram Samuha, established in the villages encouraging local men and women by inculcating leadership skills amongst them. The executive body of these Gram Samuhas is the Gram Vikas Committee (GVC), a body that has 50% women members. During the preparatory phase of the project, the GVCs deliberated over aspects of the proposed research and intervention in their villages. Members of village panchayat (council) also participated in most discussions. A powerful idea is that communities were directly involved in developing the content of the proposed research.

Based on this feasibility study, the intervention was revised in several ways. First, the item list was amended by removing unpopular items (e.g., mirror), adding new items (e.g., ventilation and basic repairs), and switching to more sustainable cleaning items. Second, the village-level commitment was swapped to a household-level pledge. And thirdly, there was learning that resulted in conducting the intervention inside the latrine rather than away from it. Lastly, a valuable outcome is that in so far as the intervention targeted female householders, this is a group of potential and actual users, who as individuals remain more receptive to using latrines compared to others.

**Fig. 2:**
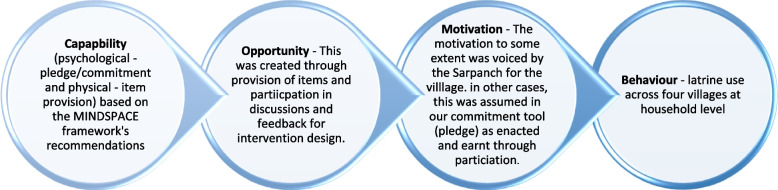
Adapted COM-B model (Capability, Opportunity, Motivation and Behaviour)

A similar household-level activity is reported by the recent Sundara Grama intervention in rural Odisha, India, which involved a household-level pledge, poster and latrine repairs [8]. Compared to this, our study is distinct in two ways. First, our household-level intervention is founded on theory from behaviour science and economics (See Fig. 2. COM-B Model) with an integrated methodical approach drawing on empirical evidence in designed stages. This adds to a mass of theorizing steeped in the behavioural science literature.

In general, behavioural change theory based approaches remain scarce in the literature. Our current pilot feasibility study will be followed by a larger project to reduce OD practice, particularly in the context of accelerated transmission of disease and the emergence of pandemics across developing countries around the world.

## Discussion

Amidst growing calls for theory informed interventions, the current study conducted a formative evaluation of a pilot behaviour change intervention, implemented in the Udaipur district of Rajasthan. These constitute tribal areas of India where latrine use and OD is an ongoing issue. Over the decades, most interventions to improve latrine use have had a modest effect [16]. Drawing on our research in Rajasthan, below some suggestions are presented by way of improvements to interventions and their chances of success in India’s rural population areas. This addresses the question “what solutions could help increase latrine use?” by way of better interventions that are feasible in light of our trialled study intervention, and the barriers identified.

Our findings provide an improve model to fit the data; with significant results in Table 2.The barriers identified and captured by the intervention include safety, relief and convenience related concerns of the villagers. Qualitative data showed that the women across the villages did not feel safe for fear of being attacked and so our study found social and house level support for latrine use. Gendered roles were pronounced in villages, more so in some than others. Given scarcity of water and poor infrastructure, common across regions and villages of Udaipur, it is the women alone who are responsible for collection of water and cleaning and maintenance of latrines.

The very idea that men could contribute to such activities was seen to be unthinkable and women who otherwise may have preferred latrine use over OD seemed to think that the latter added to their burden. The infrastructure had been in bad condition in past years and did not encourage latrine use. The villager’s emotions, perhaps underlying feelings of safety or lack of it when using latrines remotely, as well as formation of perceptions and attitudes, persist as factors [2–4, 24]. In this context, the current study demonstrated a commitment to latrine use by villagers. In terms of broader concerns about intervention implementation, it was found that fear among the villagers was also reflected in the manner in which they interacted with the study team. None of them appeared to be speaking candidly and it was apparent that they were afraid of backlash from the authorities in case they stated anything that was seen as politically incorrect.

Results support the view that there are ongoing infrastructure and resource issues and sociocultural drivers underlying motivations to use latrine use relative to alternatives such as OD [21, 39–44]. In the visited villages, interventions to change behaviour should work with local government and support in the form of local leaders like the Sarpanch. Evidence suggests that the faciliative role of district authorities, local leaders and influencers and representatives, is extremely important [15]. Whilst villagers can be emotionally engaged [44] their behaviour can be episodic and may not last [45] – making behavioural change unsustainable in the longer term.

Current study findings may not be generalizable in larger populations due to demographic variation, socio-cultural idiosyncrasies and other relevant differences across India’s vast rural landscape. With regard to improving latrine use, challenges exist for similar future interventions that may adopt randomized controlled trials (RCTs) to improve latrine use [40, 46]. First, blinding both participants and investigators can be challenging. Our study design involves prescribed procedures and elements of judgement by investigators. This makes blinding particularly important for potential RCT interventions [46]. Second, with regard to contamination, it is plausible to ask whether participants practice the intervention in such a way as to influence behaviour in the control arm [47]. Ideally, interventions should moderate for bias effects in the field, as well as the issue of courtesy bias [1]. The presence of social desirability bias highlights the tendency to record errors given the data collectors or in cases, villagers who desire identity with the intervention, may play along. The result includes ambiguous and unreliable outcomes. The current study involved training and discussions with stakeholders, yet there remains no failsafe measure, the risk should be recognised. Thirdly, another difficulty arises when scaled-up programs in the field do not deliver the same benefits that were measured in smaller efficacy trials. Additionally, there is parallel risk of imperfect compliance by villagers [48].

We propose that in measuring the primary outcome of latrine use and OD, behaviour indicating adherence should be captured effectively. The current study recommends using average latrine use per person within a household. One method of measuring this is to use an electronic occupancy scanner. This draws on the Passive Latrine Use Monitor (PLUM) approach, which has worked in rural India [49]. The technology is accurate, however, a shortcoming recognized by studies is that it does not permit distinction between household users and nonusers (such as visitors) [6]. Whilst the current study is limited to a feasibility project, a future cluster trial would consider contamination as a concern.

Also, in cases where the intracluster correlation is high, a stepped wedge trial would be suitable. A large sample can be obtained and organised on the ground with local government and agencies, like Seva Mandir, which provides NGO frameworks and social capital and reach. The Swachh Bharat Mission was India’s ambitious sanitation campaign to end OD. It ended in 2019 with success in several areas of the nation. In particular, the rural areas of Rajasthan were declared open defecation free (ODF) in March 2018 [28]. The Swachh Survekshan annual cleanliness review initiated by India in 2016 is a countrywide monitoring tool, which reported a marked improvement^3^ across regions. In the 2019 report Rajasthan demonstrated a marked improvement, whilst the Udaipur area showed innovation and initiative.

In this area, the current study shows that to improve sanitation and hygiene a sustainable strategy includes building toilets that have low water requirement alongside independent surveys to estimate OD levels [50]. Under the Swachh Bharat Mission, no fixed latrine design is mandated. Rather, EcoSan or dry pit structures may suffice given climatic conditions, resource constraints and water dynamics. This provides the opportunity to compare with other latrine systems, in terms of uptake and adherence to beneficial and healthy behaviours. On asking about Eco-san toilets, villagers expressed clear preference for flush toilets since they had seen them in the city and in use by extended family living there. Further research may focus on comparisons of results across users of different latrine technology and types, across rural settings. To monitor ongoing hygiene behaviours the Swachh Bharat Mission provides inspection and cross-verification at several levels, namely, at block, district, state and national levels.

Our results also emphasize the role of emotions and how these were factors influencing perceptions of use. The use of theory informed visual cues and physical amenities (see Additional file 1: Appendix 1 household level posters) that work to nudge behaviour and develop new habits is encouraged [26, 27].

Theory informed behaviour change interventions are tools for building capacity at village and household levels, to contribute sustainable habits and behaviours across rural households. The current study – in part designed to identify factors to optimize our pilot intervention [44] - enabled refinement on several fronts [41, 42]. First, in preliminary stages of identifying the intervention, amendments were made to the item list by removing unpopular items (e.g. mirror) adding new items (e.g. ventilation and basic repairs) and switching to more sustainable cleaning items. This improvement in design was largely a result of the feedback loops built into the intervention development process of doing research. Second, the village-level commitment was swapped to a household-level pledge. This change was an improvement that resulted from carrying out the present study. Third, there was learning in so far as the intervention was conducted inside, rather than away from the latrine. That is, the intervention was designed around the availability and accessibility concerns of villagers, rather than a remote poster on the corner of every village alley. Exposing proximity to eliciting stimuli as a significant dimension of design. Finally, in applying the refined pilot intervention, our design was reoriented around recognition that female householders, were a group that was more receptive to using latrines according to current study. A significant finding to benefit future designs.

### Limits

Since this study has a feasibility and design focus, some limits are noted. The design improvement and revisions remain informative and limited to similar conditions in rural settings around South East Asia. Due to cultural variations across countries, changes in design may be required in so far as this is recommended as a consideration of contingent factors [12, 41]. Social, psychological and structural factors may constraint and vary in their influence on choices to own and use latrines [43]. Further research on larger scales, or in other rural settings may consider concerns raised with regard to design and content, including latrine user identification and population level awareness, as well as demographic variations discussed in this regard.

## Conclusion

Further research into sanitation and hygiene is needed to reduce disease associated practices of OD and its hazards in rural parts of India. Our study supports and encourages latrine use in rural India. Behavioural Interventions can be revised, refined and adapted to improve their impact on uptake of latrine use. The intervention was developed based on views of key stakeholders and was guided by the COM-B model, MINDSPACE framework, with the outcome seemingly feasible and acceptable. The developed intervention is suitable for design-adjusted implementation to a range of Countries that continue to suffer from disease, poverty and resource constraints, with rising infant mortality and poor participation and empowerment of women in some rural contexts [4, 5, 7, 51].

### Supplementary Information

**Additional file 1.**

## Data Availability

The data that support the findings of this study are available from [the international Initiative for Impact Evaluation (3ie)] but restrictions apply to the availability of these data, which were used under license for the current study, and so are not publicly available. Data are however available from the corresponding author upon reasonable request and with permission of [the international Initiative for Impact Evaluation (3ie)]. “Study participants or the public were not involved in the design, or conduct, or reporting, or dissemination plans of our research.“ For details contact: Professor Ivo Vlaev. Ivo.vlaev@wbs.ac.uk – Warwick Business School.

## Footnotes

1 Semi-pukka are households made of a mix between hard durable material, such as cement and less durable materials like clay or bamboo. Pukka houses are made of durable high-grade materials (e.g., stone or cement).

2 Some contributing factors identified for low latrine use were poor water availability, incomplete construction, complete but poor construction and attitudinal barriers. These were observed on house visits along with conversations that established use frequency.

3 The figures at ULB level are available at Swach Sarvekshan Survey, the World’s Largest Cleanliness Survey. 2019. Available online: http://www.swachhsurvekshan2020.org/Images/SS2019%20Report.pdf (accessed on 27 February 2021).
