# Supplementary material for: Development of a behaviour change intervention to promote sanitation and latrine use in rural India
Source: BMC Public Health. 2023 Nov 6;23:2176. doi: 10.1186/s12889-023-17061-0 (PMC10629081; doi:10.1186/s12889-023-17061-0)
Supplement: Supplementary file 1 — Additional file 1. [file 12889_2023_17061_MOESM1_ESM.docx]

**Additional file 1.**

**APPENDIX 1**

**Commitment Intervention**

On the bases of our research findings, we concluded that latrine use bares some similarities with another behavioural domain known as ‘treatment adherence’ (completing a program of treatment which could include taking medication, exercise program, diet, and using a device such as latrine). Our formative research has highlighted several key barriers to adherence. There is little positive feedback around benefits adherence; in fact, initial feedback might be negative (e.g. side effects in medication, or discomfort/smell/fear in latrine use). Also, there are not enough tangible rewards for adherence - the benefits are invisible. Individuals also cannot grasp the long-term impact of non-adherence (including OD) discounting the future (hypothetical illness) in favour of the power of now (e.g. the pit is a visible reminder of the hassle that is required to empty it one day, the necessity to bring water and clean the latrine is immediate, unpleasant effort). People also find it hard to resist temptation (hassle-free OD) and it is easy to cheat oneself (hence why self-reporting defecation behaviour is often inaccurate). Lack of routine (habit) to use the latrine is another key barrier. On the social side, people expressed a difficulty in committing to latrine use, and would often miss to act and not feel guilty.

Our solution is a commitment tool, which aimed to facilitate the creation of a new habit. The Commitment principle in the MINDSPACE framework postulates that “we seek to be consistent with our public promises, and reciprocate acts”. The intervention utilizes several commitment techniques that tap into those psychological processes by also utilizing Messenger, Incentives, and Ego mechanisms of change.

(1) A poster located in the household. The intervention contains several components, some of which have been successfully piloted in the context of treatment adherence (see the images below). The poster intervention includes:

- Habit formation intervention to help individuals (or households) develop an adherence routine. Participants in the intervention group will receive a poster with a printed calendar upon it. They will be asked to track their latrine use by placing stickers (smiling face) on the days they defecate in the latrine. The feedback provided by the stickers is psychological tool recognised as integral to behaviour change, while the smiling face provides a positive reward reinforcing the action. The habit intervention also asks patients to sign a behavioural contract indicating the context (place, time, and action) surrounding when they expect to take their medication. For instance, a patient may write on the poster a promise to use the latrine every morning after waking up (action). Through repeated pairings, this context should become a trigger for patients to use the latrine, which persists after the poster ceases. Thus, the intervention contain all necessary ingredients involved in habit development: trigger (context), action (routine), and reward (reinforcement).
- The public “Promise Contract” signed by the household deploys a behavioural commitment device, a behaviour change technique which dials up saliency around the role of family/loved ones in supporting behaviour change.
- The poster shows a photo of the eyes and the face of a family member or loved one, which are a simple prime to encourage ‘good’ behaviour; for example, the photo of the children which reminds the parents that latrine use is also about the healthy future of their offspring.

(2) This poster is accompanied by a second commitment device: public pledge at village meetings and signing a pledge that is stuck at the wall in the village hall so everybody can see who committed to use their toilet. The poster/letter should also contain a photo of the family. This could be a version of the poster below, but without the self-report calendar and the eyes.

(3) A gift given to the household for small improvements of their experience using the latrine, which should trigger desire to reciprocate our kindness by using the latrine.

Illustrations of those three intervention components are presented below.

***(1) Household level poster***


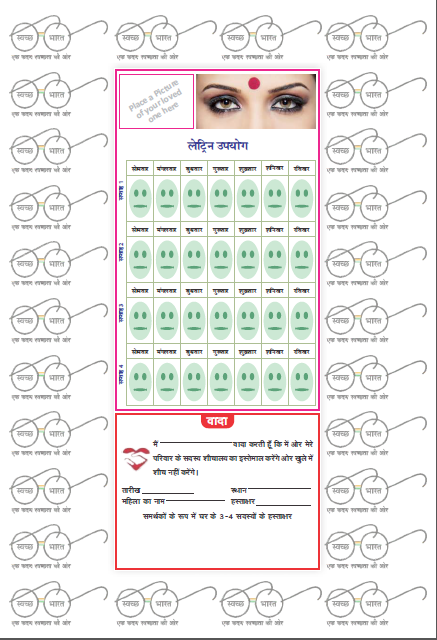


**Messenger:** Picture of loved ones acts as a motivational tool.

**Social pressure:** The presence of eyes keeps our behaviour in check as we feel like being watched.

**Incentive for habit formation:** Self-report calendar. Households affix sticker to report adherence for each day. Thus they also get rewarding feedback to their own behaviour.

**Commitment:** behavioural contract is a commitment device that helps keep behaviour on track to a goal. It keeps the commitment at the forefront of the household’s attention.

***(2) Village level poster***


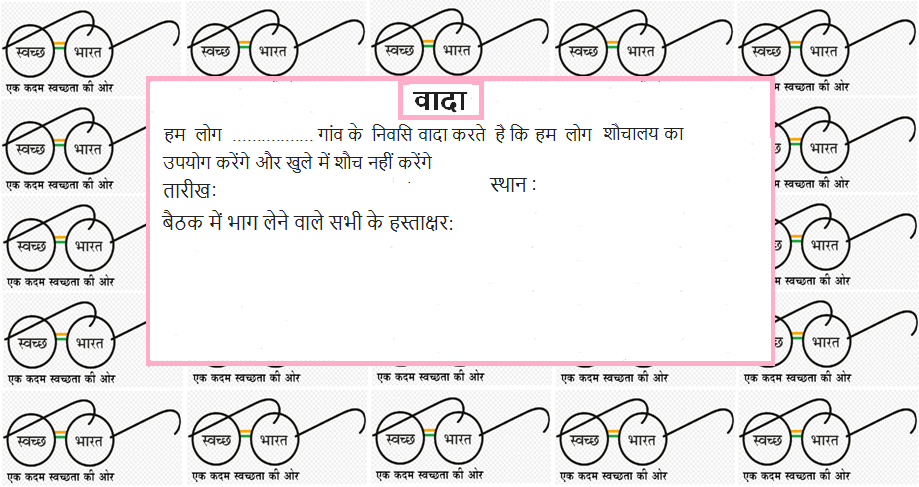


***(3) Commitment booklet for the small improvements gift***

**APPENDIX 2**

Pilot plan and “script”

**Outline of how we will conduct the pilot and suggested script**

**A. General Plan**

- Conduct: **(i) focus groups**; and **(ii) individual HH discussions**
- The HH for (ii) individual HH discussions would not be invited to (i) focus groups to preserve the independence of the household approach

**B. Initial approach**

- Introducing pilot:
  - *“We are Seva Mandir and are trying to understand why people may not use a latrine and what could be done to help make latrines more usable”*
  - *“We know that latrine use can be hard and there are many reasons why people may not use a latrine”*
  - *“What are the main reasons you don’t always use a latrine”*
- Introducing intervention:
  - *“We are trying an idea to encourage latrine use and we want to get your honest feedback about whether it would help or whether it wouldn’t really make a difference”*

**C. Commitment**

- Introduce the commitment, including showing the different elements, and ask if people are willing to sign up for the commitment:
  - *“We are looking for people to pledge to use the latrine for 30 days”*
  - *“This won’t be for everyone, we understand some barriers are still too difficult, would you be interested in signing this pledge to commit to use the latrine for the next 30 days?”*
- Show ***either poster or booklet (not both)***

**Poster:** Take them through each element of the poster and fill in the commitment together:

- - *“The poster would showcase your commitment to using the latrine. There is space to place a picture of your family to showcase who you are protecting by no longer defecating in the open. It also includes a calendar where you can place a sticker for each day that you/your family has been OD free”*
  - *Would you be willing to hang the poster and where would you place it?*

**Booklet:** Take them through each page of the booklet, filling it in together:

- - *“The booklet has questions about 1) why using the latrine is important to you and your family 2) what the barriers are 3) how you can overcome these barriers.*
  - *Which elements of the booklet do you agree and disagree with?*
  - *Are there any elements that you think are missing from the booklet?*

**D. Items**

- Explain why we introduce the items:
- *“We understand there are important barriers to using the latrine. It may, however, be possible to overcome some of these with some small changes/improvements”*
- *“Please can you tell us again what the main barriers are - and we will think about these relate best to the items we have”*
- Introduce each item:
  - *“I am going to introduce each item, say why it may help and at the end we ask you to chose* ***two*** *items and why you choose these specific ones”*
- Items:
  1. Bucket - jug: Perception of too much water use
  2. Cleaning brush - cleaning product - holder: Fear of maintenance
  3. Hand wash: Worries about dirtiness
  4. Light: Difficult to use at night
  5. Air freshener: Worries of smells
  6. Mirror: Normalizes people to going into the toilet
  - *Which two items do you think will help you overcome the barriers you are facing to latrine use”?*
  - *Are the two items too few, enough, too many?*
  - *Are there any items that you are missing”*
  - *Where would you place the items*
  - *When do you think you would use the item if at all*

**E. Conduct feasibility and acceptability survey (modified APPEASE)**

**APPENDIX 3**

**Significant Variables**

| Independent variables | Type of variable | Coding | Significance | How it is affecting the behaviour |
| --- | --- | --- | --- | --- |
| Village | Nominal | A-1, B-2, C-3, D-4 | Significant variable |  |
| Caste | Nominal | SC-1, ST-2, OBC-3, Gen-4 | Not a significant variable |  |
| APL/BPL | Ordinal | BPL-1, APL-2 | Significant variable | BPL families are more likely to use latrine |
| Type of latrine | Nominal | Pit-1, Septic-2, Ecosan-3, Others-4 | Not a significant variable |  |
| House type | Ordinal | Kuchha-1, Semi pucca-2, Pucca-3 | Not a significant variable |  |
| Electricity connection | Ordinal | No-1,Yes-2 | Not a significant variable |  |
| Difficulty in getting drinking water | Ordinal | No-1,Yes-2 | Not a significant variable |  |
| Difficulty in getting water for consumption | Ordinal | No-1,Yes-2 | Not a significant variable |  |
| Land holding size | Ordinal | Landless-1, Marginal-2, Small-3, Medium-4 | Not a significant variable |  |
| Difficulty in getting water for cultivation | Ordinal | No-1,Yes-3, NA=2 | Significant variable | Difficulty in getting water for cultivation reduces the use of latrine |
| Source of income | Nominal | Agriculture-1, Farm/Nonfarm labor-2, Livestock-3, Private business-4, Salaried-5, skilled-6, Others-7 | Not a significant variable |  |
| Monthly expenses of HH | Ordinal | less than 5000-1, between 5000 to 10000-2, between 10000-20000-3, more than 20000-4 | Significant variable | As the expenses of HH increases the chance of using latrine also increases |
| Main earning member of HH | Ordinal | Other than head-1, Head of HH-2 | Not a significant variable |  |
| Seen any advertisements/posters/other IEC material that aimed at motivating to use latrines | Ordinal | No-1,Yes-2 | Not a significant variable |  |
| Position of latrine in HH | Ordinal | More than 10m distance-1, Less than 10m-2, Inside house-3 | Significant variable | The HH having latrine inside the house are more likely to use latrine |
| Funding for latrine | Nominal | Self-1, Government funded-2, NGO -3, combination of self contribution and Govt/NGO fund -4,Others-5 | Not a significant variable |  |
| cost HH incur in construction of latrine | Ordinal | less than 5000-1, between 5000 to 10000-2, between 10000-20000-3, more than 20000-4 | Significant variable | As the expense incurred by the HH on the construction of latrine increases, the use of latrine also increases |
| Year of construction of latrine | Ordinal | Before 2012-1, 2012-2, 2013-3,2014-4,2015-5,2016-6,2017-7 | Not a significant variable |  |
| you need to defecate, is the latrine easy to find and visible | Ordinal | No-1,Yes-2 | Not a significant variable |  |
| Place of defecation do your family members find most comfortable/convenient | Ordinal | Latrine=3, no such difference/ some members find latrine comfortable and some OD =2,Open Defecation=1 | Significant variable | People who say latrine use is most comfortable/convenient are more likely to use latrine |
| find latrine use convenient | Ordinal | No-1,Yes-2 | Significant variable | People who say latrine use is not convenient are less likely to use latrine |
| social pressure to use latrine | Ordinal | No-1,Yes-2 | Significant variable | People who admit there is a social pressure to use latrine are less likely to use latrine. This may be due to the person using latrine may not have facing any pressure to use latrine where as the HH those do not use latrine are facing pressure to use latrine. It shows there exist a village level pressure to use latrine. |
| Perception about villagers use of latrines | Ordinal | All of them =1, Most of the villagers=2 (60-90%), Some people in villages=3 (40-60%), Few people in village=4 (less than 40%), No idea=5 | Significant variable | It shows people who do not use latrine have opinion that higher proportion of HH use latrine compared to HH who use latrine |
| Emotion-angry | Ordinal | No-0,Yes-1 | Not a significant variable |  |
| Emotion-happy | Ordinal | No-0,Yes-1 | Significant variable | People who are happy thinking about latrine are more likely to use latrine |
| Emotion-anxious | Ordinal | No-0,Yes-1 | Not a significant variable |  |
| Emotion-disgust | Ordinal | No-0,Yes-1 | Not a significant variable |  |
| Emotion-safe | Ordinal | No-0,Yes-1 | Significant variable | People who feel safe thinking about latrine are more likely to use latrine |
| Emotion-relief | Ordinal | No-0,Yes-1 | Significant variable | People who feel relief thinking about latrine are more likely to use latrine |
| Financial-gain | Ordinal | No-1,Yes-2 | Not a significant variable |  |
| Financial-loss | Ordinal | No-1,Yes-2 | Not a significant variable |  |

**APPENDIX – 4**

**Qualitative Survey Questions**

1. Do you have a latrine in your household?
2. Is this latrine functional?
3. What type of latrine is it?
4. Who uses the latrine?
5. Who cleans the latrine?
6. How much water is required for each use?
7. Who brings the water for use?
8. Is there enough water for household consumption and latrine use?
9. Is the experience of using a latrine different from OD? Which do you prefer and why?
10. Why, in your opinion, is OD being discouraged?
11. Why is the government constructing toilets for every household?
12. How much money was spent by your family in constructing the latrine? What was the total cost of construction?
13. What is stopping you from using the latrine despite having one in your household?
14. Does every household in your village have latrines?
15. Which are the households which still do not have latrines and why?
16. Do people in your village prefer OD or use latrines?
17. What, in your opinion, could be done to change people’s habits from OD to latrine use?

**Appendix – 5**

| 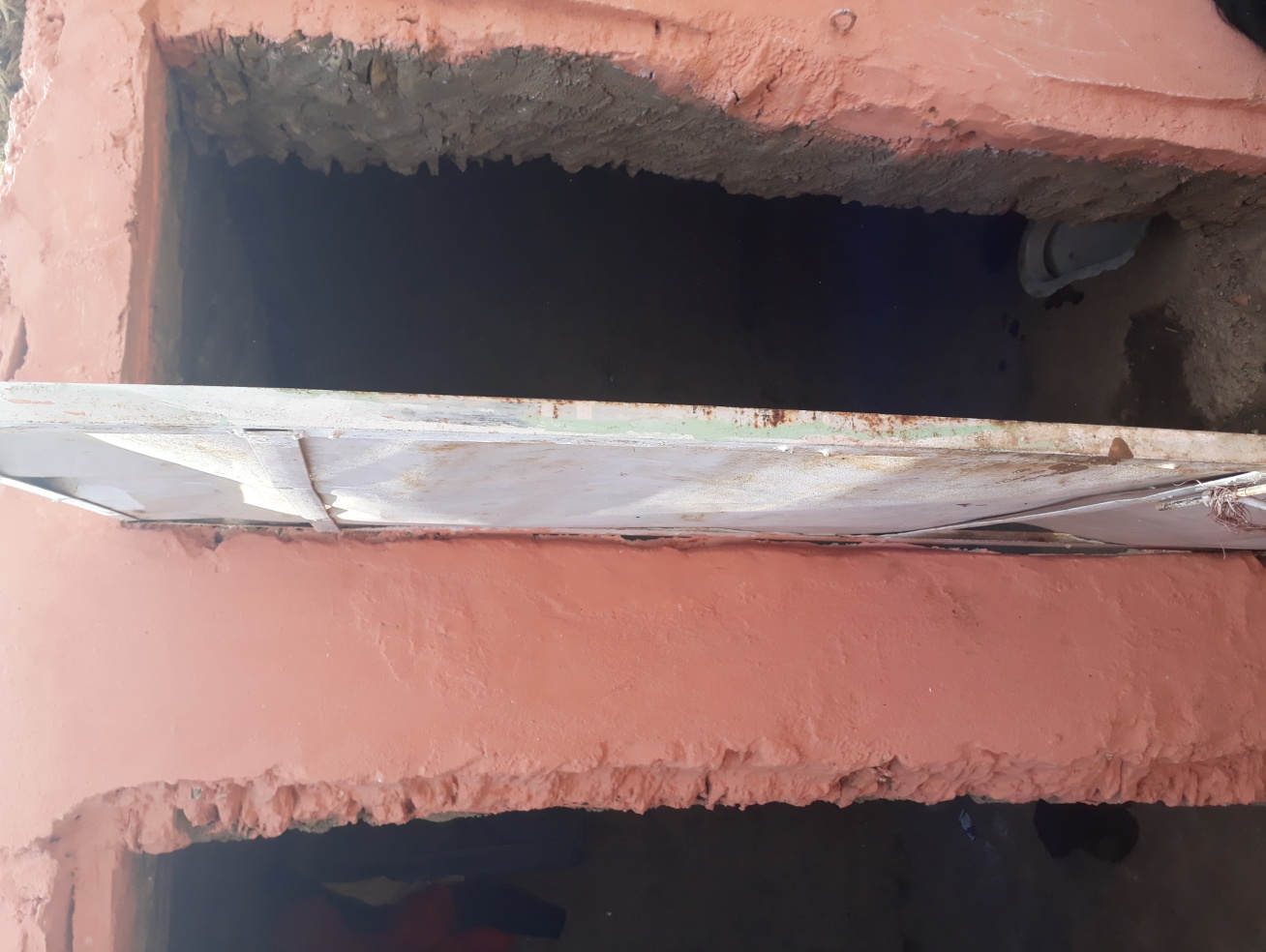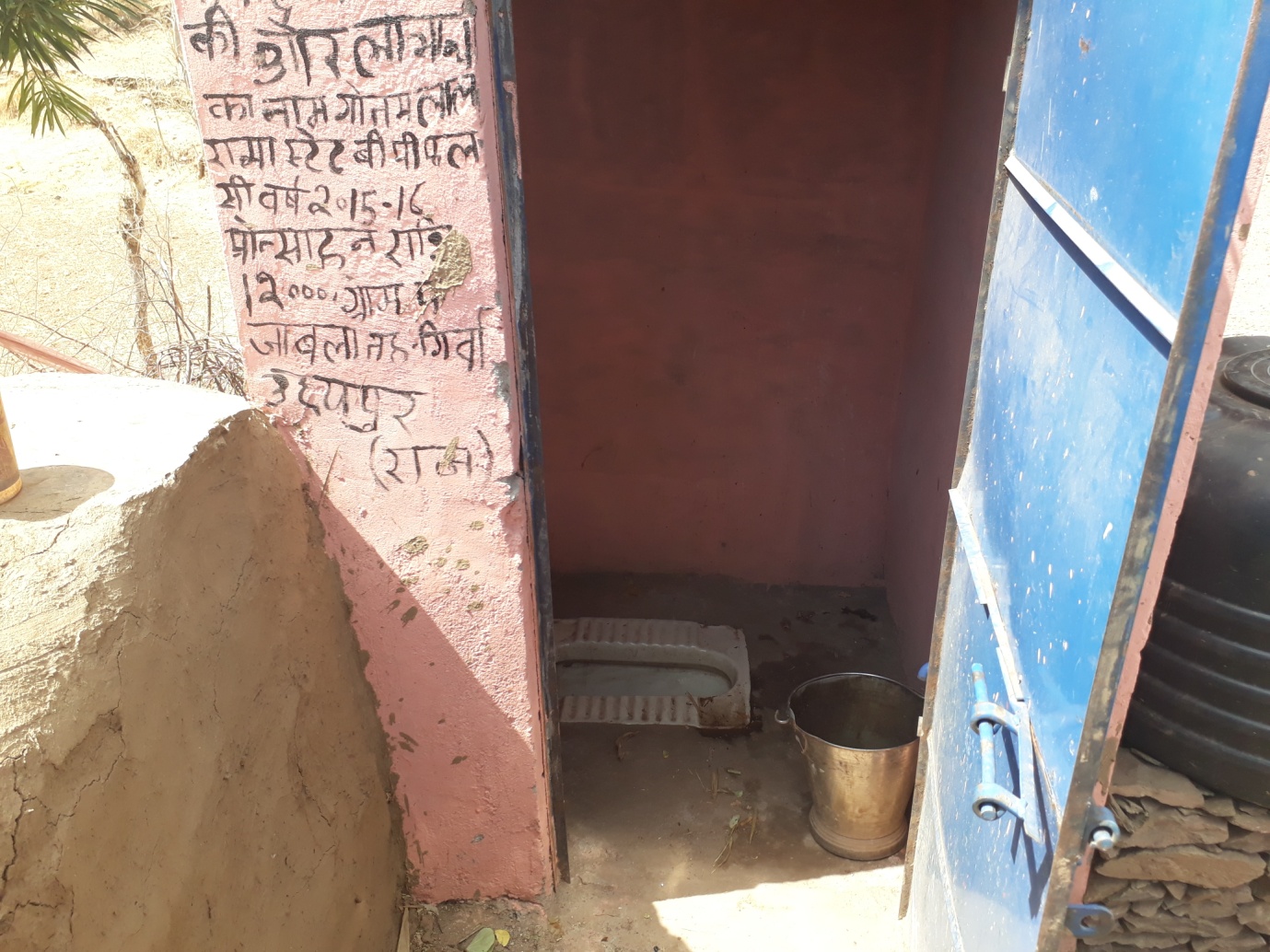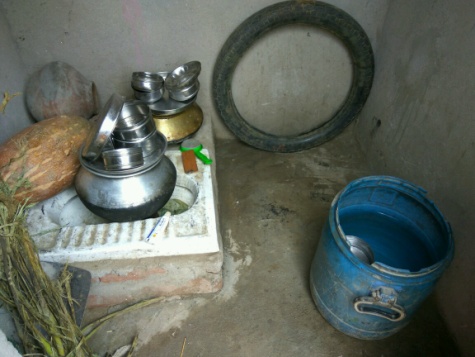 |
| --- |
| 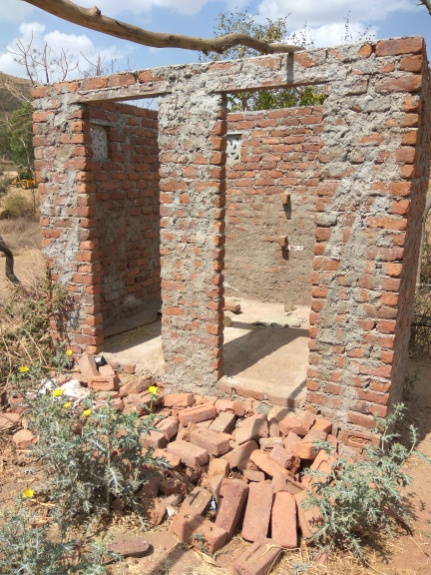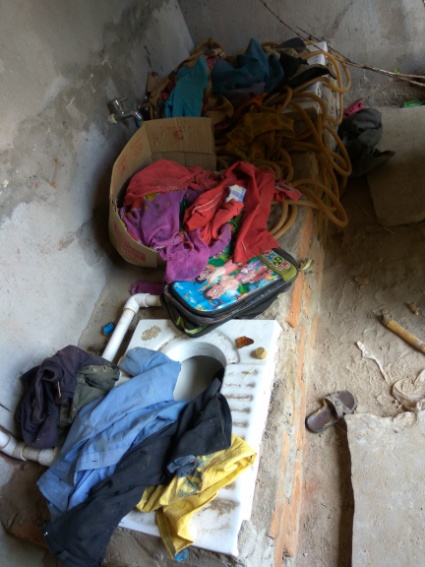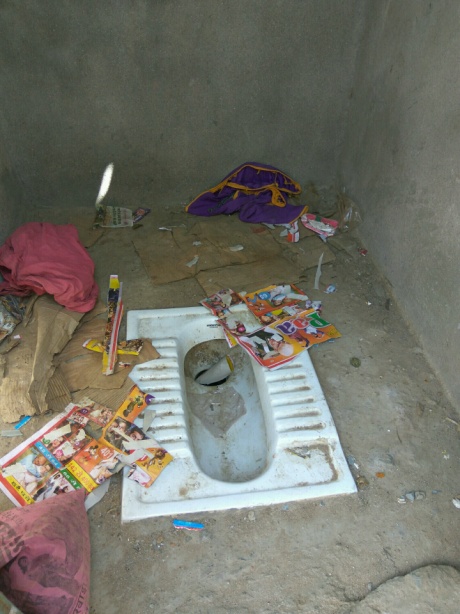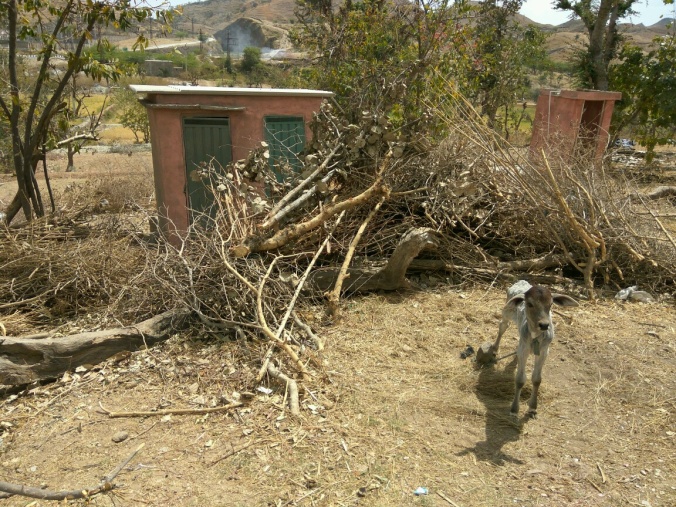  Field note – the infrastructure and associated provisions are not attractive and mostly induce negative emotional responses in many villagers. Hence, convenience and safety were associated concerns found in our statistical results.  **Appendix – 6**  Quantitative Survey Tool  **GENERAL INFORMATION**   \| Interviewer Group (Gr A, Gr B, GrC, GrD) \|  \| 1. Village name \|  \| \| \| --- \| --- \| --- \| --- \| --- \| \| 1. Household ID ***(To be filled by Researcher)*** \|  \| \| \| \| \| 1. Age of Respondent \|  \| 1. Gender of Respondent (Male, Female) \|  \| \| \| 1. Religion (Hindu-1, Muslim-2, Christian-3, Jain -4, Sikh- 5, Don’t want to reveal-6) \|  \| 1. Caste of HH (SC-1, ST-2, OBC-3, Gen-4, Cannot say= 5) \|  \| \| \| 1. Status of HH (APL-1/BPL-2) \|  \| 1. Number of members in HH \| Male Members \|  \| \| Females Members \|  \| \| 1. How many members in your HH are earning member \|  \| 1. How many members in your HH are literate \|  \| \| \| 1. How many members in the HH are pursuing education \|  \| 1. How many members in the HH own a mobile phone \|  \| \| \| 1. Does the HH have constructed a latrine \|  \|  \|  \| \| \|  \|  \|  \|  \| \|   **B. TOILET OBSERVATION (The surveyor must observe himself or herself and fill in this section)**   \| 1. Type of latrine \| Pit=1, Eco San=2, Septic=3, Others=4 \|  \| \| --- \| --- \| --- \| \| 1. Can you see a water seal inside the latrine? \| Yes=1, No=0 \|  \| \| 1. Is the platform of the latrine wet? \| Yes=1, No=0 \|  \| \| 1. Can you see a water container inside the latrine? \| Yes=1, No=0 \|  \| \| 1. Is there an arrangement to store larger quantities of water in the latrine? \| Yes=1, No=0 \|  \| \| 1. Are any cleaning supplies (such as brush, harpic or acid) visible? \| Yes=1, No=0 \|  \| \| 1. Can you see feces inside the latrine? \| Yes=1, No=0 \|  \| \| 1. Is any foul odor emanating from the latrine? \| Yes=1, No=0 \|  \| \| 1. Is there a vent pipe from the latrine pit? \| Yes=1, No=0 \|  \| \| 1. From seeing the latrine, can you say that the latrine is being used? \| Yes=1, No=0 \|  \|   **C. HOUSEHOLD DETAILS**   \| 1. **House ownership**   Own=1, Rented=2 \| 1. **House type**   Kucha=1, Pucca=2, Semi Pucca=3 \| 1. **No of rooms in the house** \| \| 1. **Does the HH have electricity connection**   Yes=1, No=0 \| \| --- \| --- \| --- \| --- \| --- \| \|  \|  \|  \| \|  \| \| 1. **Source of drinking water**   Piped water=1, Tube well/hand pump=2, surface (pond/river/well/canal)=3, Others=4 specify \| \| \| 1. **Does the HH face any difficulty in getting enough drinking water for family need?**   Yes=1, No=0 \| \| \|  \| \| \|  \| \| \| 1. **Source of water for other consumption**   Piped water=1, Tube well/hand pump=2, surface (pond/river/well/canal)=3, Others=4 specify \| \| \| 1. **Does the HH face any difficulty in getting enough water for other consumption use**   Yes=1, No=0 \| \| \|  \| \| \|  \| \| \| 1. **What is the most prominent illness among HH members?** **(Multiple)** Diarrhea=1, typhoid fever=2, infectious hepatitis=3, worm infestations=4, cough=5, TB=6, malaria=7, others=8 specify, No illness= 9 \| \| \| \| \| \|  \| \| \| \| \|   **D. INCOME OF HH**   \| **Land Ownership and Family Overview** \| \| \| \| --- \| --- \| --- \| \| 1. Does the HH have cultivable land? \| Yes=1, No=0, **If 0 skip to 5** \|  \| \| 1. How much land does the HH possess? \| In Bigha \|  \| \| 1. How many crops does the HH cultivate annually? \| In number \|  \| \| 1. Does the HH face water problems in cultivation? \| Yes=1, No=0 \|  \| \| **Household Income Details** \| \| \| \| 1. What is the main source of income? \| Code 1 \|  \| \| 1. What is the secondary source of income? \| Code 1 \|  \| \| 1. How much is the monthly expenses of the HH? \| In INR \|  \| \| 1. What is/are the time period during which members fall sick in the year (multiple) \| Summer=1, Rainy=2, Spring=3, No specific season=4, No season=5 \|  \|   Code 1: Agriculture=1, Farm/Non farm labor=2, Livestock rearing / dairy=3, skilled work (masonry / carpentry / plumbing / pottery / weaving / craftsman / blacksmith / tailoring / barber / sweet making / washer man/ electrician/ mechanic/cook and others)=4, any business=5, salaried job in govt/ private =6, Shop job=7, , others=8 specify  **E. DECISION MAKING IN HH**   \| 1. Who is the main earning member in the HH? \| Head of HH=1, Other member=2 \|  \| \| --- \| --- \| --- \| \| 1. Who takes day today decision in your house? \| Only head of HH=1, Other member=2, head of the HH and other members=3, others=4 \|  \| \| 1. Are women members consulted during following decisions? \| Usually consulted=1, sometimes consulted=2, not consulted=3 \|  \| \| 1. Children’s education \|  \|  \| \| 1. Children’s marriage \|  \|  \| \| 1. Healthcare decisions \|  \|  \| \| 1. House construction/renovation \|  \|  \| \| 1. Major investments and savings \|  \|  \|   **F. LATRINE SECTION**   \| 1. What would you say is the main benefit of using a latrine \| Open ended \|  \| \| --- \| --- \| --- \| \| 1. Which of the among is the benefit of using latrine (Multiple) \| Keeps the environment clean=1, good practice=2, Privacy=3, safe and convenient=4, no benefit=5, Others=6 \|  \| \| 1. Who made decision to construct the latrine? \| Code 2 \|  \| \| 1. Have you seen any advertisements/posters/other IEC material that aimed at motivating you to use latrines in your village? \| Yes=1, No=0 \|  \| \| 1. Has anyone in the community asked you to use construct and use a latrine? \| Yes=1, No=0, **If No go to 7** \|  \| \| 1. Who has asked you to construct and use a latrine? \| Sarpanch/Ward member=1, School teacher=2, AWW =3, Religious people=4, Government person=5, NGO people=6, others=7, specify \|  \| \| 1. Where is the latrine in the house? \| Inside the house=1, Within 10 m distance from house=2, more than 10m from house=3 \|  \| \| 1. Who funded for the construction of latrine? \| Self-1, Government funded-2, NGO -3, combination of self contribution and Govt/NGO fund -4,Others-5 specify \|  \| \| 1. What is the approximate cost HH incur in construction of latrine? \| in INR \|  \| \| 1. How many years back was the latrine constructed? \| Number of years \|  \| \| 1. What is the type of latrine? \| Pit =1, latrine with septic tank=2, Eco san =3, Others=4 specify \|  \| \| 1. Who does the cleaning of latrines in your household? **(multiple option)** \| Code 2 \|  \| \| 1. Who brings the water that is used in the latrine? \| Code 2 \|  \| \| 1. Has the latrine pit got filled and required cleaning? \| Yes=1, No=0**, If 0 skip Q20** \|  \| \| 1. How many months did it take to fill the latrine pit? \| In months \|  \| \| 1. Who cleaned the latrine pit when it got filled? \| Our family members=1, Other people =2, **If 1 go to 18** \|  \| \| 1. Which caste people did the latrine pit cleaning? \| (SC-1, ST-2, OBC-3, Gen-4, Cannot say= 5) \|  \| \| 1. Did you face any difficulty in emptying the latrine pit? \| Yes=1, No=0 **if no skip to section G** \|  \| \| 1. What was the difficulty? \| None of our household members can/want to do this= 1  We could not find anyone else to do it=2  Outside people are expensive=3  Others= 4 (specify) **Go to Section G** \|  \| \| 1. How much time it will take to fill the latrine pit? \| In months \|  \|   Code2 Head of household=1, Spouse of head=2, Parent of head=3, Son / daughter of head=4, Brother/Sister of Head=5, Spouse of brother /sister of head=6, Spouse of married son / daughter=7, Grandchild of Head of Household=8 Other relative=7   \| **G. Latrine usage** \| \| \| \| --- \| --- \| --- \| \| 1What is the latrine use of all HH members \| If all members use latrine=1, If all members go for OD=2, If members use latrine and go for OD=3 **If 1 go to G1, If 2 Go to G2, If 3 go to G3** \|  \| \| **G.1 Only latrines** \| \| \| \| 1. How do you feel when you think about using a latrine (multiple) \| Relief=1, Anxious=2, Safe=3, Disgust=4, Happy=5, Angry=6, Others=7 \|  \| \| 1. When you need to defecate, is the latrine easy to find and visible? \| Yes=1, No=0 \|  \| \| 1. Which place of defecation do your family members find most comfortable/convenient? \| Latrine=1, Open Defecation=2, no such difference=3, some members find latrine comfortable and some OD =4 \|  \| \| 1. What are the benefits of using a latrine? \| Keeps the environment clean=1, good practice=2, privacy is protected=3, Using latrine is safe and comfortable=4, Others=5 specify, No benefit=6 \|  \| \| 1. What are the benefits of OD? \| Good environment=1, no bad smell=2, it is comfortable=3, use of water is less=4, you do not have to defecate inside the house=5, less or no cost=6, Others=7 specify, No benefit=8 \|  \| \| 1. What are the problems with latrine use? \| Very dirty smell =1, Sitting in latrine is not comfortable =2, not enough water for using latrine=3, Defecating inside the house is harmful=4, pit is small and using regularly can fill it early=5, Others=6, specify, No benefit=7 \|  \| \| 1. What are the problems with OD? \| Going outside is not safe=1, Going unusual time is difficult=2, have to go long distance=3, privacy is compromised=4, Others=5, specify, No benefit=6 \|  \| \| 1. Do you find latrine use convenient ? \| Yes=1, N0=0 \|  \| \| 1. Do you get bad smell while using latrine? \| Yes=1, N0=0 \|  \| \| 1. Is there any social pressure to use latrine? \| Yes=1, N0=0 **If no go to 12** \|  \| \| 1. What are those social pressures? \| All the villagers have decided to use only latrine=1, Government workers tell to use latrine, NGO worker tell to use latrine=3, all neighbors use latrine=4, privacy is compromised if going for OD =5, Others=6, specify (multiple options) \|  \| \| 1. Is there any financial gain in using latrine? \| Yes=1, N0=0 **If no go to 14** \|  \| \| 1. What is the gain? \| Open ended \|  \| \| 1. Is there any financial loss in using latrine? \| Yes=1, N0=0 **If no go to 16** \|  \| \| 1. What is the loss? \| Use to purchase cleaning material=1, Pit cleaning is required=2, others=3, specify \|  \| \| 1. Do most of the people in village use latrine? \| All of them =1, Most of the villagers=2 (60-90%), Some people in villages=3 (40-60%), Few people in village=4 (less than 40%), No idea=5 \|  \| \| 1. Will your family members continue to use latrine in future \| Yes=1, N0=0 \|  \| \| 1. Do you think latrine use give higher status among other villagers? \| Yes=1, N0=0 \|  \| \| 1. Do you feel safe due to latrine use? \|  \|  \| \| 1. Do you feel proud of the fact that you are using a latrine? \| Yes=1, N0=0 **Go to G4** \|  \| \| **G.2 Only OD** \| \| \| \| 1. How do you feel when you think about using a latrine (multiple) \| Relief=1, Anxious=2, Safe=3, Disgust=4, Happy=5, Angry=6, Others=7 \|  \| \| 1. When you need to defecate, is the latrine easy to find and visible? \| Yes=1, No=0 \|  \| \| 1. Which place of defecation your family members find comfortable/convenient? \| Latrine=1, Open Defecation=2, no such difference=3, some members find latrine comfortable and some OD =4 \|  \| \| 1. What are the benefits in using latrine? \| Keeps the environment clean=1, good practice=2, privacy is protected=3, Using latrine is safe and comfortable=4, Others=5 specify, No benefit=6 \|  \| \| 1. What are the benefits of OD? \| Good environment=1, no bad smell=2, it is comfortable=3, use of water is less=4, you do not have to defecate inside the house=5, less or no cost=6, Others=7 specify, No benefit=8 \|  \| \| 1. What are the problems with latrine use? \| Very dirty smell =1, Sitting in latrine is not comfortable =2, not enough water for using latrine=3, Defecating inside the house is harmful=4, pit is small and using regularly can fill it early=5, Others=6, specify, No benefit=7 \|  \| \| 1. What are the problems with OD? \| Going outside is not safe=1, Going unusual time is difficult=2, have to go long distance=3, privacy is compromised=4, Others=5, specify, No benefit=6 \|  \| \| 1. Do you find latrine use convenient? \| Yes=1, N0=0, Not used=2 \|  \| \| 1. Is there any social pressure to use latrine? \| Yes=1, N0=0 **If no go to 11** \|  \| \| 1. What are those social pressures? (multiple) \| All the villagers have decided to use only latrine=1, Government workers tell to use latrine, NGO worker tell to use latrine=3, all neighbors use latrine=4, privacy is compromised if going for OD =5, Others=6, specify (multiple options) \|  \| \| 1. Is there anything preventing you from gaining access to a latrine? \| Yes=1, N0=0 \|  \| \| 1. What are the issues? \| Open ended \|  \| \| 1. Is there any financial gain in using latrine? \| Yes=1, N0=0 **If no go to 15** \|  \| \| 1. What is the gain? \| Open ended \|  \| \| 1. Is there any financial loss in using latrine? \| Yes=1, N0=0 **If no go to 17** \|  \| \| 1. What is the loss? \| Use to purchase cleaning material=1, Pit cleaning is required=2, others=3, specify \|  \| \| 1. Do most of the people in village use latrine? \| All of them =1, Most of the villagers=2 (60-90), Some people in villages=3 (40-60), Few people in village=4 (less than 40), No idea=5 \|  \| \| 1. Will you and your family members be interested in using latrine in the future? \| Yes=1, N0=0 \|  \| \| 1. Do you think latrine use give you higher status in the village \| Yes=1, N0=0 \|  \| \| 1. Do you think it is normal to go for OD \| Yes=1, N0=0 \|  \| \| 1. Where do you defecate? \| Own farm=1, Other farm=2, other place=3 \|  \| \| 1. Do you go for defecation in a group or go alone? \| Group=1, alone=2, **if 2 go to 24** \|  \| \| 1. Who goes with you for defecation? \| Friends=1, family member=2, Others=3 specify \|  \| \| 1. Are there separate places for men and women to go for open defecation? \| Yes=1, No=0 \|  \| \| 1. Do you face any problem if you have to go for open defecation in day time? \| Yes=1, No=0, If yes specify \|  \| \| 1. Do you face any problem if you have to go for open defecation during night? \| Yes=1, No=0, If yes specify \|  \| \| 1. Do you face any problem if you have to go for open defecation when you are unwell? \| Yes=1, No=0 \|  \| \| 1. Do you face any problem if you have to go for open defecation during monsoons? \| Yes=1, No=0 \|  \| \| 1. Is the respondent is a woman? (observation) \| Yes=1, No=0 , **If 2 go to 31** \|  \| \| 1. Do women members in the family face any problem if they go for open defecation during menstruation period? \| Yes=1, No=0, \|  \| \| 1. Do you feel safe due to latrine use \| Yes=1, No=0 \|  \| \| 1. What can motivate you to use latrine \| Open ended **(go to G4)** \|  \| \| **G.3 OD and Latrine** \| \| \| \| 1. How do you feel when you think about using a latrine (multiple) \| Relief=1, Anxious=2, Safe=3, Disgust=4, Happy=5, Angry=6, Others=7 \|  \| \| 1. When you need to defecate, is the latrine easy to find and visible? \| Yes=1, No=0 \|  \| \| 1. Which place of defecation your family members find most comfortable/convenient? \| Latrine=1, Open Defecation=2, no such difference=3, some members find latrine comfortable and some OD =4 \|  \| \| 1. What are the benefits in using latrine? \| Keeps the environment clean=1, good practice=2, privacy is protected=3, Using latrine is safe and comfortable=4, Others=5 specify, No benefit=6 \|  \| \| 1. What are the benefits of OD? \| Good environment=1, no bad smell=2, it is comfortable=3, use of water is less=4, you do not have to defecate inside the house=5, less or no cost=6, Others=7 specify, No benefit=8 \|  \| \| 1. What are the problems with latrine use? \| Very dirty smell =1, Sitting in latrine is not comfortable =2, not enough water for using latrine=3, Defecating inside the house is harmful=4, pit is small and using regularly can fill it early=5, Others=6, specify, No benefit=7 \|  \| \| 1. What are the problems with OD? \| Going outside is not safe=1, Going unusual time is difficult=2, have to go long distance=3, privacy is compromised=4, Others=5, specify, No benefit=6 \|  \| \| 1. Do you find latrine use convenient? \| Yes=1, N0=0, not used=2 \|  \| \| 1. Do you find any bad smell while using latrine? \| Yes=1, N0=0, not used=2 \|  \| \| 1. Is there any social pressure to use latrine? \| Yes=1, N0=0 **If no go to 12** \|  \| \| 1. What are those social pressures? \| All the villagers have decided to use only latrine=1, Government workers tell to use latrine, NGO worker tell to use latrine=3, all neighbors use latrine=4, privacy is compromised if going for OD =5, Others=6, specify (multiple) \|  \| \| 1. Is there anything preventing you from gaining access to a latrine? \| Yes=1, N0=0, **if 0 go to 14** \|  \| \| 1. What are the issues? \| Open ended \|  \| \| 1. Is there any financial gain in using latrine? \| Yes=1, N0=0 **If no go to 16** \|  \| \| 1. What is the gain? \| Open ended \|  \| \| 1. Is there any financial loss in using latrine? \| Yes=1, N0=0 **If no go to 18** \|  \| \| 1. What is the loss? \| Use to purchase cleaning material=1, Pit cleaning is required=2, others=3, specify \|  \| \| 1. Do most of the people in village use latrines? \| All of them =1, Most of the villagers=2 (60-90), Some people in villages=3 (40-60), Few people in village=4 (less than 40), No idea=5 \|  \| \| 1. Will you and your family members be interested to use only a latrine in the future? \| Yes=1, N0=0 \|  \| \| 1. Do you think latrine use give higher status among other villagers? \| Yes=1, N0=0 \|  \| \| 1. Do you think it is normal going for OD? \| Yes=1, N0=0 \|  \| \| 1. Where do your family members go for defecation? \| Own farm=1, Other farm=2, other place=3 \|  \| \| 1. Do your family members go for defecation in a group or go alone? \| Group=1, alone=2, **if 2 go to 25** \|  \| \| 1. Who goes with you for defecation? \| Friends=1, family member=2, Others=3 specify \|  \| \| 1. Are there separate places for men and women to go for open defecation? \| Yes=1, No=0 \|  \| \| 1. Do you face any problem if you have to go for open defecation in day time? \| Yes=1, No=0, if 1 specify \|  \| \| 1. Do you face any problem if you have to go for open defecation during night? \| Yes=1, No=0, if 1 specify \|  \| \| 1. Do you face any problem if you have to go for open defecation when you are unwell? \| Yes=1, No=0, if 1 specify \|  \| \| 1. Do you face any problem if you have to go for open defecation during monsoons? \| Yes=1, No=0, if 1 specify \|  \| \| 1. Is the respondent is woman (observation) \| Yes=1, No=0, **If 0 skip to 32** \|  \| \| 1. Do women members in the family face any problem if they go for open defecation during menstruation period? \| Yes=1, No=0 \|  \| \| 1. Have you used latrine yesterday? \| Yes=1, No=0 \|  \| \| 1. Is there any specific time/situation you go for OD \| Open ended \|  \| \| 1. What steps can motivate you to use latrine \| Open ended \|  \| \| 1. Do you have to think where to go for defecation every time you feel pressure? \| Pre-decided=1, spontaneous=2 \|  \| \| 1. Do you feel safe due to latrine use? \| Yes=1, No=0 \|  \| \| **G.4 Migration use** \|  \|  \| \| 1. Does any male member of family migrate during the year? \| Yes=1, No=0, If **No end of interview** \|  \| \| 1. In which period does the member migrate? \| 12 months multiple option \|  \| \| 1. For how many months the member migrate? \| Number (1-12) \|  \| \| 1. What is the reason for migration? \| Earning =1, Others=2, specify \|  \| \| 1. Where does the member stay during migration? \| Employer provide=1, rented by himself=2, relative=3, others=4 \|  \| \| 1. Is there latrine facility where the member stays? \| Yes=1, No=0, Do not know=3 \|  \| \| 1. Where does the member defecate during stay? \| OD=1, Latrine=2, no specific place=3 **End of interview** \|  \|   **H. HH WHICH DO NOT HAVE LATRINE FACILITY**   \| 1. Why have you not constructed toilet in your house? (Multiple) \| Do not feel the need=1, do not have enough money=2, no space=3, no support from Govt /NGO=4, others=5 specify \|  \| \| --- \| --- \| --- \| \| 1. What in your view will be the cost for construction of toilet in your opinion? \| **In INR** \|  \| \| 1. Have you applied for latrine construction under TSC/SBM? \| Yes=1, No=0, **if no go to 5** \|  \| \| 1. Which year did you apply? \| **Year** \|  \| \| 1. Have you applied for latrine construction under any NGO scheme? \| Yes=1, No=0, **if no go to 7** \|  \| \| 1. Which year did you apply? \| **Year** \|  \| \| 1. How many members in your household are interested in using latrine? \| **Number** \|  \| \| 1. Do you/your family members face any issues while going for OD? \| Yes=1, No=0 \|  \| \| 1. Do you feel female members feel unsafe while going for OD? \| Yes=1, No=0 \|  \| \| 1. Do you feel female members privacy compromised while going for defecation? \| Yes=1, No=0 \|  \| \| 1. Does your village have any community latrine? \| Yes=1, No=0, **If 0 go to 17** \|  \| \| 1. Do your HH members use community latrine? \| Yes=1, No=0 \|  \| \| 1. Do you have to pay for using community latrine? \| Yes=1, No=0, **if 0 go to 15** \|  \| \| 1. How much you have to pay per use? \| **In INR** \|  \| \| 1. How far is the community latrine from your house? \| In meters \|  \| \| 1. What are the reasons for not using community latrine? (Multiple) \| Not clean=1, not convenient=2, distance from house=3, have to give money=4,others=5, specify \|  \| \| 1. Do you think there are any benefits in using latrines? \| Yes=1, No=0 \|  \| \| 1. Comfortable \|  \|  \| \| 1. Convenient \|  \|  \| \| 1. Safety for women \|  \|  \| \| 1. Privacy is maintained \|  \|  \| \| 1. Easy for old and disabled person \|  \|  \| \| 1. Latrine use improves health \|  \|  \| \| 1. If there is anything you want to say about latrine usage? \| Open Ended ( End of interview) \|  \| |
